# Radiomics in Oncology: A Practical Guide

**DOI:** 10.1148/rg.2021210037

**Published:** 2021-10-01

**Authors:** Joshua D. Shur, Simon J. Doran, Santosh Kumar, Derfel ap Dafydd, Kate Downey, James P. B. O’Connor, Nikolaos Papanikolaou, Christina Messiou, Dow-Mu Koh, Matthew R. Orton

**Affiliations:** From the Department of Radiology, Royal Marsden Hospital NHS Foundation Trust, Sutton, England (J.D.S., D.a.D., K.D., N. P., C.M., D.M.K.); Institute of Cancer Research, 15 Cotswold Road, Sutton SM2 5NG, England (S.J.D., S.K., J.P.B.O., N. P., C.M., D.M.K., M.R.O.); and Computational Clinical Imaging Group, Champalimaud Foundation, Centre for the Unknown, Lisbon, Portugal (N.P.).

## Abstract

Radiomics refers to the extraction of mineable data from medical imaging and has been applied within oncology to improve diagnosis, prognostication, and clinical decision support, with the goal of delivering precision medicine. The authors provide a practical approach for successfully implementing a radiomic workflow from planning and conceptualization through manuscript writing. Applications in oncology typically are either classification tasks that involve computing the probability of a sample belonging to a category, such as benign versus malignant, or prediction of clinical events with a time-to-event analysis, such as overall survival. The radiomic workflow is multidisciplinary, involving radiologists and data and imaging scientists, and follows a stepwise process involving tumor segmentation, image preprocessing, feature extraction, model development, and validation. Images are curated and processed before segmentation, which can be performed on tumors, tumor subregions, or peritumoral zones. Extracted features typically describe the distribution of signal intensities and spatial relationship of pixels within a region of interest. To improve model performance and reduce overfitting, redundant and nonreproducible features are removed. Validation is essential to estimate model performance in new data and can be performed iteratively on samples of the dataset (cross-validation) or on a separate hold-out dataset by using internal or external data. A variety of noncommercial and commercial radiomic software applications can be used. Guidelines and artificial intelligence checklists are useful when planning and writing up radiomic studies. Although interest in the field continues to grow, radiologists should be familiar with potential pitfalls to ensure that meaningful conclusions can be drawn.

*Online supplemental material is available for this article.*

Published under a CC BY 4.0 license.

## Graphical abstract

**Figure:**
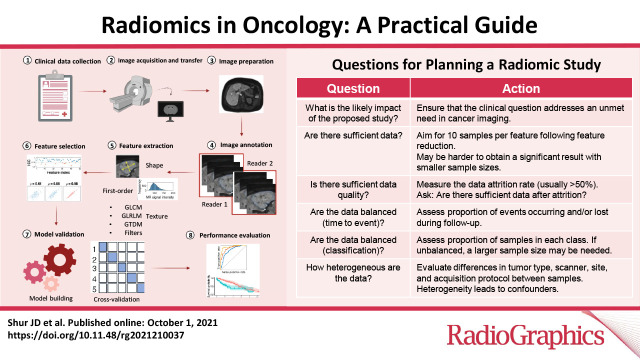
Created with BioRender.com

## SA-CME LEARNING OBJECTIVES

*After completing this journal-based SA-CME activity, participants will be able to:*

■ List the main applications of radiomic studies in oncology.
■ Understand the use of image pre-processing, segmentation, and validation in radiomic studies.
■ Describe the main radiomic feature classes and how they are calculated.

## Introduction

Radiomics refers to the extraction of mineable high-dimensional data from radiologic images (1–3) and has been applied within oncology to improve diagnosis and prognostication (4,5) with the aim of delivering precision medicine. The premise is that imaging data convey meaningful information about tumor biology, behavior, and pathophysiology (6) and may reveal information that is not otherwise apparent to current radiologic and clinical interpretation.

The radiomic workflow involves curation of clinical and imaging data and is a stepwise process involving image preprocessing, tumor segmentation, feature extraction, model development, and validation (7). It is a field that requires input from individuals in many disciplines, including radiologists, imaging scientists, and data scientists. Features are derived at single (usually pretreatment) or multiple (eg, δ radiomics) time points and can be applied to the whole spectrum of imaging data.

**Figure 1.**
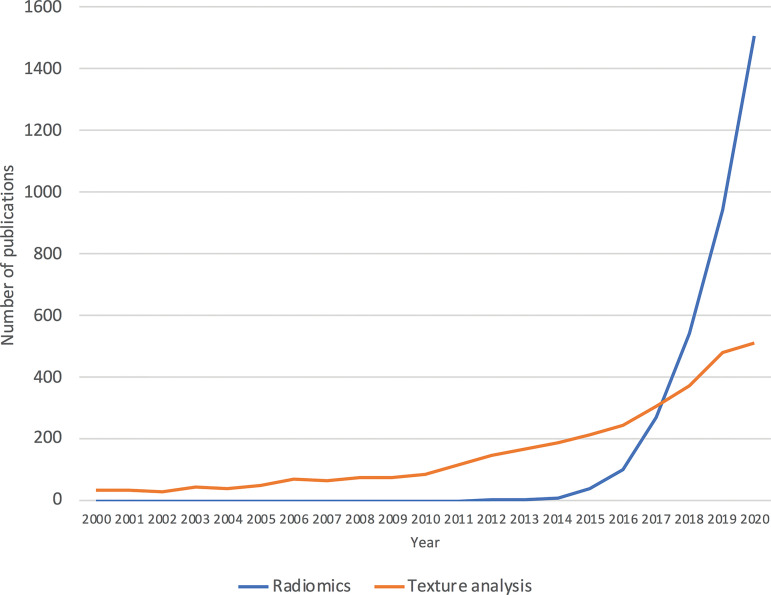
Graph shows the number of publications per year since 2000 that contain the terms *radiomics* and *texture analysis* in PubMed *(www.pubmed.gov)*. Since first being coined in 2012, the term *radiomics* in the literature has demonstrated an exponential increase, numbering over 1500 publications in 2020 alone. The term *radiomics* has overtaken *texture analysis* in publications in PubMed, indicating a shift toward *radiomics* as the preferred term in the research literature.

Although many of the concepts of image feature extraction have been around for decades (8), research output in the field has grown exponentially, with over 1500 publications in 2020 containing the term *radiomics* (Fig 1). With increasing interest in the field, there is a need for an understanding of the radiomic workflow and its challenges and limitations so that robust conclusions may be drawn (9). The purpose of this article is to provide a practical hands-on guide for implementing radiomic studies in oncology and a glossary of terms for readers less familiar with the topic (Table 1).

**Table 1:**
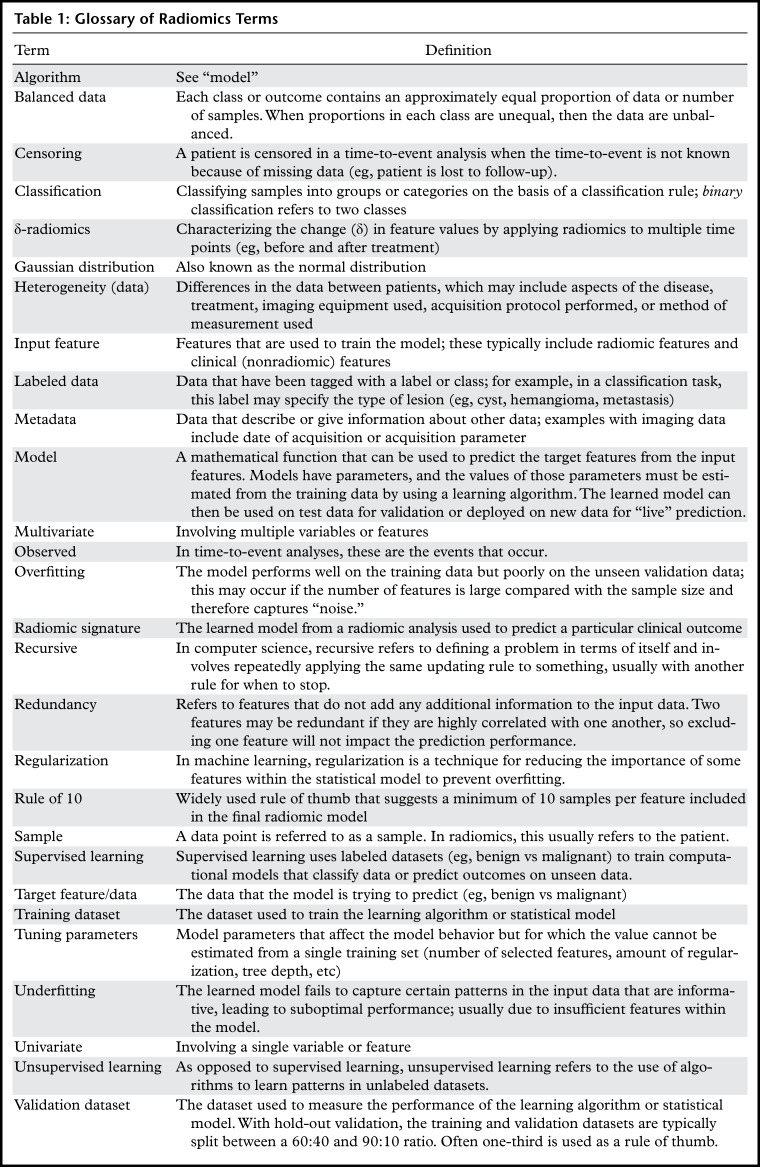
Glossary of Radiomics Terms

## Applications in Oncology

Radiomic studies in oncology are usually either *(a)* classification tasks or *(b)* prediction of clinical outcomes by using a time-to-event analysis. Classification involves dividing a population into categories. Examples include benign versus malignant, genomic status, tumor stage, and presence of metastases, among many others. Predictive models use clinical outcomes to stratify patients into different risk groups on the basis of the risk of occurrence of clinical endpoints, such as overall or disease-free survival, and are assessed by using a time-to-event analysis.

**Figure 2.**
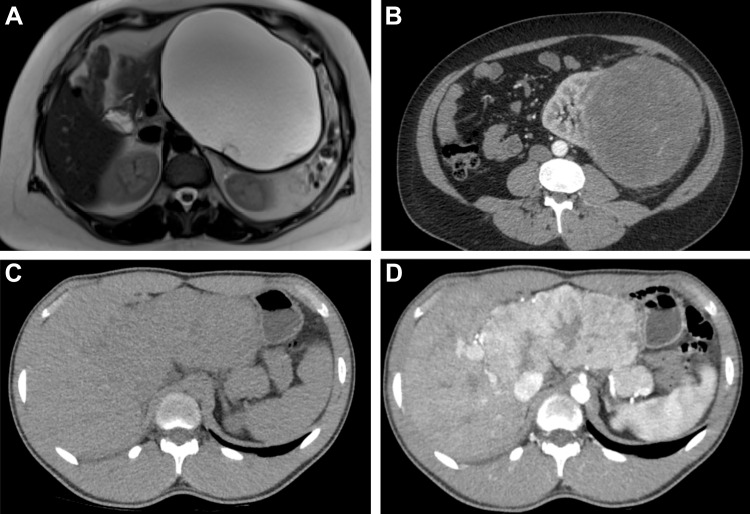
Variations in tumor heterogeneity from less to more heterogeneous are demonstrated in these abdominal masses. **(A)** Axial T2-weighted MR image in a 40-year-old woman shows a large unilocular cystic lesion in the pancreas that appears to have uniform high signal intensity (SI), with only minor nonenhancing peripheral septa and a smooth border. This appearance is typical for a mucinous cystadenoma. After surgical resection, no invasive malignancy was found. **(B)** Axial CT image shows a partly heterogeneous mass in the left kidney, which appears well defined and contains predominantly homogeneous bland-appearing tissue with streaks of vascularity. This was found to be a spindle-cell sarcoma after surgical resection. **(C, D)** Axial nonenhanced **(C)** and contrast-enhanced **(D)** CT images of a fibrolamellar hepatocellular carcinoma clearly show the heterogeneous nature of this malignant tumor, with irregular vascular enhancing tissue surrounding a less-vascular central component. Contrast-enhanced imaging is often used in radiomic analyses and is useful to help highlight vascularity and spatial heterogeneity, a determinant of tumor behavior and resistance to therapy that is not readily apparent without contrast material.

These applications are guided by the notion that radiomic data convey information about tumor biology (1). For example, radiomic features may reflect temporal and spatial heterogeneity (Fig 2), which is known to be a key determinant of tumor behavior and resistance to therapy (10). Thus, radiomics has the potential to act as a “virtual biopsy” and, unlike standard biopsies, uses noninvasive imaging that permits analysis of the whole tumor (rather than a focal sample) and can be applied more easily at multiple time points for disease monitoring, offering potentially important diagnostic information related to disease evolution.

## Planning a Radiomic Study

When planning a radiomic study, it is worth asking basic questions (Table 2) to assess feasibility and likelihood of success. At our institution, we find a radiomic study proforma useful when assessing proposed studies (Appendix E1). As with any research study, a radiomic study should have a testable hypothesis that should address a relevant clinical question, usually with the aim of meeting an unfulfilled need in cancer management.

**Table 2:**
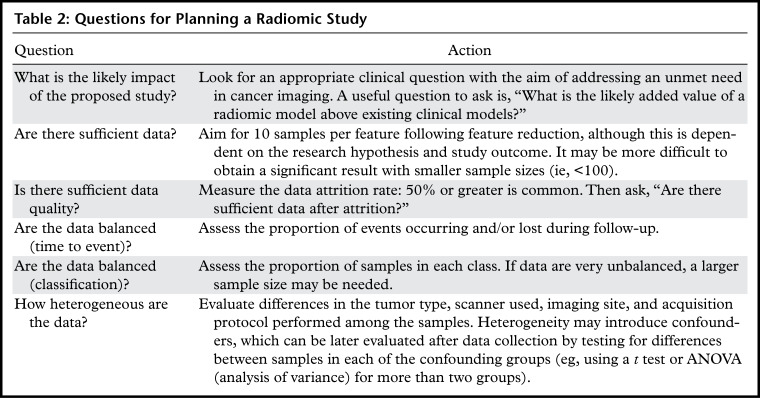
Questions for Planning a Radiomic Study

A key consideration is to determine the availability of sufficient data to support the development of a *radiomic signature* (defined as the learned model from a radiomic analysis used to predict a particular clinical outcome). As a rule of thumb for binary classification studies, one should aim to obtain 10–15 samples per feature in the final radiomic signature. This can vary between studies but is a useful guide when embarking on a new study (11,12). If the class sizes are unequal, the rule should be applied to the smaller class (13). As radiomics is data driven, it may not be possible to know in advance how many features will be included in the final model, since feature selection methods are typically applied before or during the model fitting process. It is also important to be aware that data attrition is common. Common reasons include data that are missing or mislabeled, failure to satisfy inclusion criteria or lost to follow-up, and poor image quality. These highlight the importance of obtaining a realistic estimate of the final sample size before embarking on a new study.

Model validation consists of measuring the predictive performance of the model by using data that were not used in fitting the model. Sufficient data should be available for validation of a radiomic model, typically around one-third of the training sample size. The one-third proportion represents a trade-off between having enough data in the training set to ensure the model has sufficient predictive power and having a large-enough test dataset to ensure the predicted performance estimate is accurate. Values used in practice are in the range of 60:40 to 90:10. For example, using the “one-third” criteria and a 10-feature model, at least 133 samples are required, where 100 are used for training and 33 for validation. Assuming an attrition rate of 50% would require a total study population of 266. This highlights the challenge required to curate datasets of sufficient size for high-quality radiomic studies.

Finally, it is important to consider whether the data are balanced. For classification tasks, balanced data are such that each class or outcome contains an approximately equal proportion of the data. When proportions are unequal, the data are unbalanced, and if they are very unbalanced then a larger sample size may be required for the model developed to be generalizable. For time-to-event analyses, the proportion of events that are observed (event time known) and censored (eg, subject leaves the study before the event occurs or the study ends before the event occurs) should be estimated.

Consideration should be given to data heterogeneity, including disease status, treatment, imaging equipment, acquisition protocol, and method of measurement. There exists a trade-off between real-world heterogeneous datasets, in which noise may mask an underlying radiomic signature, and well-controlled homogeneous datasets that are less noisy but have lower generalizability. An assessment of data heterogeneity is performed by evaluating how similar the study design and inclusion criteria are compared with what is encountered in clinical practice. This will assist in assessing both the chance of success and also whether a follow-up real-world study would be required to establish a clinically useful signature.

Once the research question and study population have been defined, one should consider collecting pilot data to help identify and mitigate potential problems before full data collection. With a representative sample of data, frequency of missing data and rates of passing inclusion criteria can be estimated. For classification studies, a pilot sample size of 12 per class has been proposed (14), but in practice this is guided by available resources and the study population. Running pilot data through the radiomic processing pipeline as early as possible enables issues to be resolved quickly, and any preliminary results may be able to guide the final sample size. For example, if a signature is detected but is not statistically significant, then it may be possible to estimate the number of samples required to obtain a significant result.

## Radiomic Workflow Overview

**Figure 3.**
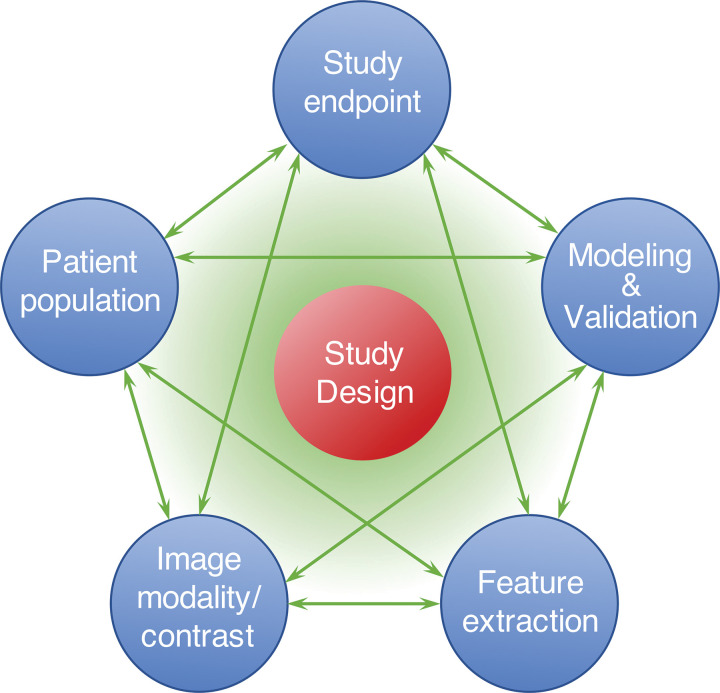
As demonstrated in this diagram, the study design arises by considering the interaction of multiple criteria or activities, including patient population, study endpoint, available imaging and/or clinical data, radiomic feature extraction methodology, and appropriate modeling and validation strategy.

**Figure 4.**
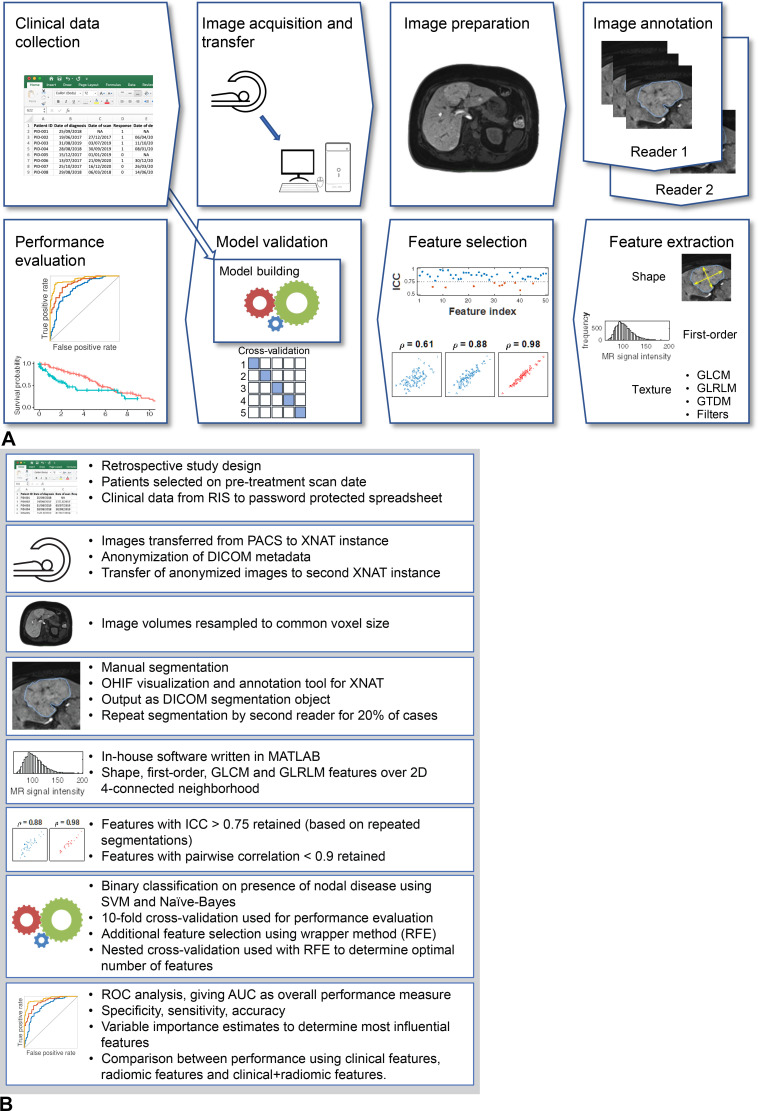
**(A)** Overview of a typical radiomic workflow that embodies the study design and details the steps involved in taking clinical and imaging inputs all the way through to the study endpoint. **(B)** Details of each stage should be clearly reported to allow meaningful interpretation, discussion, and critique of the study findings. The workflow used in Doran et al (15) is illustrated. The authors investigated the utility of radiomics from multivendor multi-parametric MRI in prediction of lymph node status in patients with breast cancer. *AUC* = area under the curve, *DICOM* = Digital Imaging and Communication in Medicine, *GLCM* = gray-level co-occurrence matrix, *GLRLM* = gray-level run-length matrix, *GTDM* = gray-tone difference matrix, *ICC* = intra-class correlation coefficient, *OHIF* = Open Health Imaging Foundation, *PACS* = picture archiving and communication system, *RIS* = radiology information system, *ROC* = receiver operator characteristic, *RFE* = recursive feature elimination, *SVM* = support vector machine, *2D* = two dimensional, *XNAT* = eXtensible Neuroimaging Archive Toolkit.

The radiomic workflow represents the combined effort of a multidisciplinary team, including data and imaging scientists and radiologists, and is subdivided into multiple tasks that are typically performed in sequence (Figs 3, 4).

## Image Acquisition

Although the majority of studies to date have used data from CT examinations, radiomic analyses can be applied to the whole spectrum of imaging data, including those from CT, PET, MRI, and US examinations. One advantage of CT and PET data are that signal intensities (SIs) are inherently quantitative. CT may also be less prone to motion artifacts seen with PET and MRI. US is more user dependent than other modalities; however, along with MRI, assessing feature stability in a test-retest experiment is feasible, as there is no radiation burden. Ultimately the choice of modality is often determined by what is available and used in clinical practice. Contrast-enhanced imaging yields information about tumor enhancement, vascularity, and heterogeneity (Fig 2) that may not be apparent without the use of contrast material but may incur a cost burden and require particular expertise (eg, ability to perform contrast-enhanced US).

Suitable imaging data that meet the study inclusion and exclusion criteria should be clearly defined. Standardized imaging protocols (ie, those that use the same vendor or scanner settings for all samples) can be used to reduce unwarranted confounders and noise (4), whereas less rigidly standardized protocols can be used to reflect real-world clinical scenarios.

Once a cohort has been identified, images should be anonymized to remove patient identifiable metadata. However, relevant nonidentifiable image data can be retained. Images should be exported as Digital Imaging and Communication in Medicine (DICOM) files by using a lossless-compressed format to avoid losing potentially informative image features. It is worth speaking with the picture archiving and communication system (PACS) team to enlist help.

## Data Curation

Nonimaging and clinical data are typically collated in a repository for analysis, and it is advisable to discuss with the institution’s or practice’s statistician or data scientist the desired format before data collection. Curation steps to identify missing or incomplete data can then be taken, along with correction of typographic errors or inconsistencies, before merging clinical and radiomic data.

## Image Preprocessing

Before feature extraction, the raw image data can be enhanced through a variety of preprocessing steps, which are summarized in Table 3. Although these may improve image quality, care should be taken as they can mask or degrade the radiomic signature and may be better mitigated against by optimizing and standardizing the image acquisition.

**Table 3:**
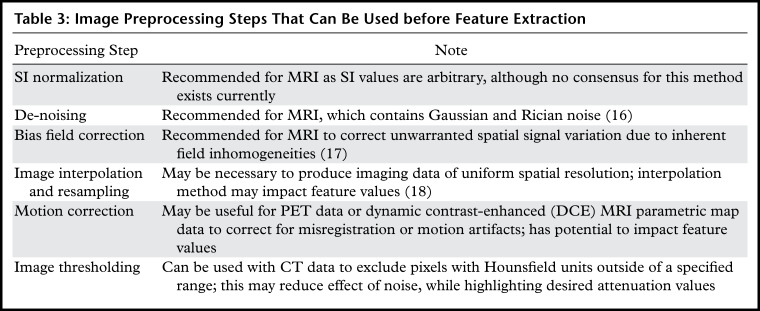
Image Preprocessing Steps That Can Be Used before Feature Extraction

Unlike at CT, units of MRI SI are arbitrary, and hence normalization of SI is recommended. Although no consensus currently exists, the z-score is a simple method and is computed by subtracting the mean SI of the region of interest (ROI) from the pixel SI and dividing the result by the standard deviation (19). Bias field correction should also be applied to correct for the spatial field inhomogeneities encountered with MRI (17). Thresholding on voxel Hounsfield units can be applied to CT data to exclude voxels that are assumed to contain noninformative tissues. For example, very low values may correspond to air within the lung and high values to bone or calcification.

As some radiomic feature values are dependent on voxel size (20), images should be resampled to a common spatial resolution for all samples (21). Linear interpolation is generally recommended (18,22).

Motion correction can be used to correct for misregistration, blurring, or motion artifacts and has been used in four-dimensional CT of lung tumors (23). However, this additional processing has the potential to impact potential radiomic information in the images. The use of motion- control techniques, such as breath holding, is advised as the effect of motion blurring on computed radiomic features is known to be feature dependent (24).

**Figure 5.**
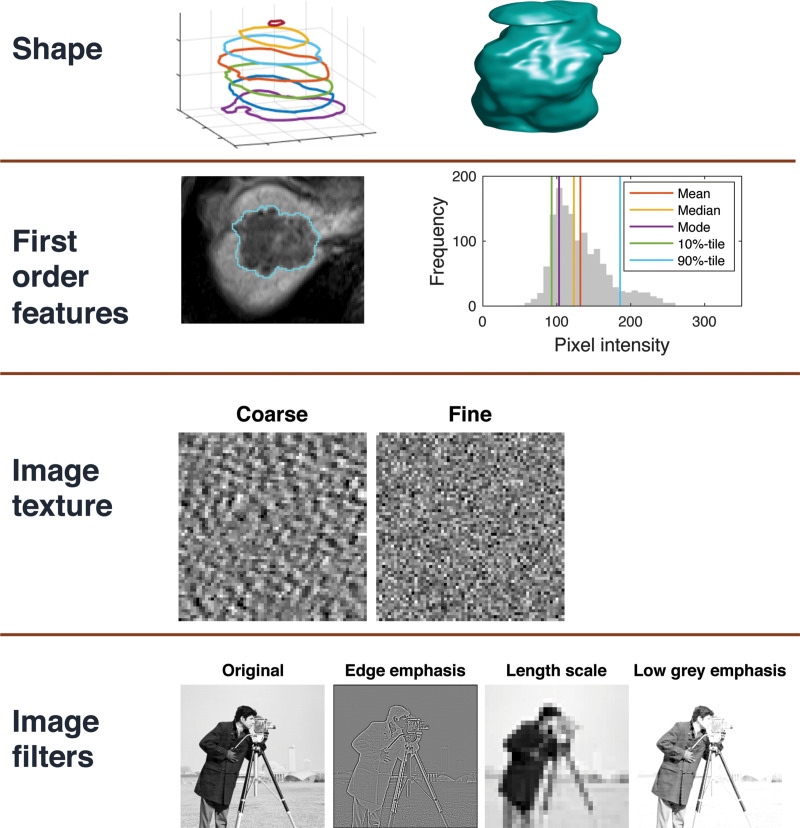
Pictorial overview of the feature classes used in most radiomic studies. Shape or morphologic features can be computed in 2D or 3D views, with 3D analysis being the recommended approach for most studies. First-order features are computed from the distribution of SIs within the ROI and include features such as the mean, median, and mode, which describe the central tendency of the data, and other features such as percentiles, skewness, kurtosis, and entropy, which describe the symmetry and heterogeneity of the distribution. Texture or second-order features consider the joint statistics of two or more voxels, so that in the coarse texture example, neighboring pairs of pixels are likely to have similar gray levels, whereas in the fine texture example, neighboring pixel values are independent. In radiologic images, the statistical dependencies between neighbors can be more complex than in these simple examples, and so features derived from the GLCM, gray-level run-length matrix (GLRLM), and other metrics can be effective for quantifying image texture. Filtering the images to emphasize edges, different length scales, or different gray levels can be used before computing texture features with the aim of sensitizing the features to a wider range of biologic correlates.

Image filtration can be used before the extraction of features as a preprocessing step to highlight particular image properties. Nonspatial filters increase or decrease the sensitivity of the radiomics features to high- or low-intensity values; examples include taking the square or exponential of the image intensities. Spatial filters increase or decrease the sensitivity of features to particular spatial properties of the image. Examples include Laplacian of Gaussian (LoG) filters, which emphasize areas of rapid change (eg, edge detection) (Fig 5) (25) and wavelet filters, which separate high- and low-spatial-frequency information. The number of radiomics features (and hence datasets) generated with image filtration can become large, so it is typical to try using unfiltered images first.

## Segmentation

Segmentation can be performed by drawing ROIs on the tumor, tumor subregions (“habitats”), or peritumoral zones, the choice guided by the research hypothesis. For example, habitat imaging aims to characterize intratumoral spatial heterogeneity by comparison of discreet functional tumor subregions (26), whereas the peritumoral zone may contain information about tumor invasion or host immune response (27). Radiation therapy tumor volume data used for treatment planning can also be used, although these may differ from ROIs specifically drawn for a radiomic analysis.

ROIs can be delineated manually, automatically, or semiautomatically in either two dimensions (2D) (single section) or three dimensions (3D) (multiple sections) (Fig 5). The choice will be determined by available resources and tumor type. Three-dimensional ROIs will capture additional information but can be time consuming to draw when manual delineation is used.

Automatic segmentation is potentially faster and more reproducible (28) and may be required for larger datasets for which manual segmentation is not feasible. However, segmentations should be checked by a radiologist to ensure accuracy. Features can be compared against those obtained after manual segmentation by using the Dice score.

When manual segmentation is used, feature stability should be assessed by performing multiple segmentations of the same tumor with either the same or a different reader performing the delineation.

## Feature Extraction

Feature extraction is the final step before model building and validation and involves computing radiomic features from each ROI that will be used in the model. Radiomic features are “handcrafted” in that the algorithms used to generate them are designed or chosen by the data scientist rather than being learned directly from the images, as is found with deep learning approaches. Consequently, it may be possible to interpret the radiomic signature obtained with handcrafted features, whereas deep learned features can suffer from limited explainability.

A wide variety of feature classes exist and are summarized in Table 4. The set of quantitative imaging features is large and is being continually updated and refined. Efforts have been made in standardization such as with the Image Biomarker Standardization Initiative (IBSI) (21), and we recommend that readers refer to this resource for an up-to-date description of features and their properties.

**Table 4:**
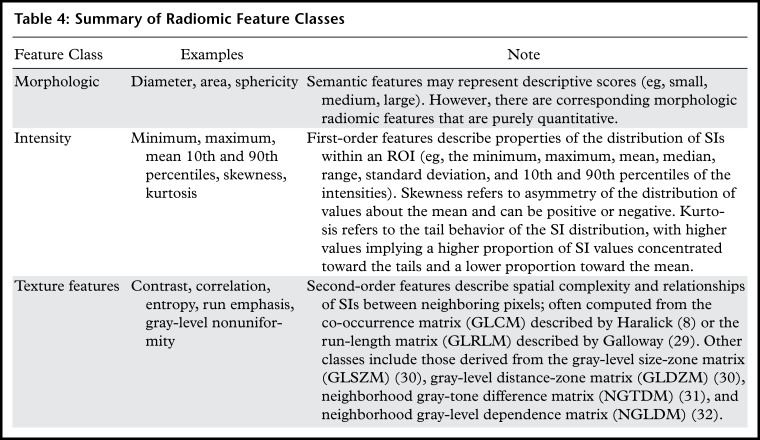
Summary of Radiomic Feature Classes

Morphologic features describe geometric properties of the lesion such as volume, diameter, surface area, and elongation. Intensity-based features, also known as first-order features, describe properties of the distribution of intensities within an ROI, where the spatial location of each voxel is ignored. First-order features can be broadly grouped into those that measure the location of the distribution (mean, median, mode, etc), those that measure the spread of the distribution (variance, interquartile range, etc), those that measure the shape of the distribution (skewness, kurtosis, etc), and other features linked to less specific properties of the voxel intensity heterogeneity (entropy, energy, etc). Imaging modalities such as MRI and US typically generate images with arbitrary intensity scaling, and if this is not consistent for all subjects it will be necessary to apply image standardization before calculating first-order features. Features such as skewness are unaffected by image standardization, as they are dependent on the shape of the distribution of intensities rather than their absolute values.

Second-order features, also known as texture features, go beyond first-order features so that the spatial locations as well as the SIs of two or more pixels are used when computing the features. For example, gray-level co-occurrence matrix (GLCM) features consider the SIs of pairs of pixels separated by a given distance and direction, while gray-level size-zone matrix (GLSZM) features consider the sizes of contiguous regions that share the same SI after discretization.

**Figure 6.**
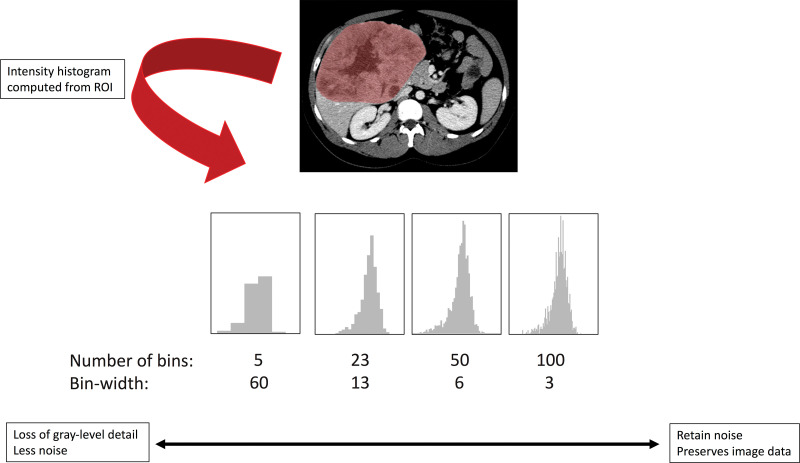
SI discretization involves assigning pixels within a given SI range to a single value or bin and is used before calculation of second-order features. In this diagram, the SI histogram is derived from an ROI encompassing a hepatic tumor with varying bin size (or bin width). Increasing the bin size or decreasing the number of bins may cause loss of image detail but reduces noise, whereas decreasing the bin size or increasing the number of bins preserves image detail at the expense of image noise. The choice of image modality and SI range will define the method of discretization.

Intensity discretization involves assigning pixels within a given intensity range to a single value or “bin” and is used before calculation of second-order features. Either the bin width or the total number of bins can be specified. Reducing the number of bins (or increasing the bin width) will lead to a loss of image detail but will remove noise (Fig 6). Conversely, increasing the number of bins (or decreasing the bin width) will retain more image detail but will also preserve image noise. Using a fixed bin size maintains the relationship of the “binned” data to the original intensity scale and can be used when the intensity scale is quantitative (such as CT and PET data). When image intensity units are arbitrary (such as with MRI data), fixing the number of bins (rather than the bin size) is recommended (21). Whichever method is used, it should be the same for all patients.

In addition to the agnostic or quantitative feature classes described, semantic features such as “spiculated” or “enhancing” can also be used as input features to a radiomics model and will be determined by visual inspection. These features will typically be categorical (eg, small, large, hyperenhancing) rather than numerical.

## Model Building

Once clinical and radiomic data are collected and curated, statistical models are fitted to predict study endpoints, such as tumor type or survival time. A typical model uses input features (including the radiomic features described previously and clinical features such as tumor markers or lymph node status) in addition to target data that the model aims to predict, such as benign versus malignant or risk of recurrence. The final performance and generalizability of models discovered from a radiomic analysis is determined by validating the model on new test data (33,34).

The hold-out method uses a training set to develop the model, and a validation set to estimate future performance on new data. To avoid biasing the model performance, the validation data should be shielded from the model training process, and the final validation only performed once. Ideally, validation data should be obtained from another institution, but this is not always possible. Splitting single-institution data into training and validation sets is often more practical and can be done randomly, temporally (by using the most recent cases as validation data), or by choosing a similar class proportion (eg, benign versus malignant) in the training and validation datasets, known as stratified sampling.

Once training and validation datasets have been established, it is important to verify that the feature distributions between the two datasets are similar. This is to ensure that any informative patterns obtained in the training data will also be present in the validation data. Independent univariate testing of each feature is typically performed, and useful tests include the Mann-Whitney *U* test (equality of the medians in the two datasets), and the Komogorov-Smirnov or Shapiro-Wilk test (equality of the distributions of the two datasets). These tests do not use the outcome data (they are referred to as unsupervised) and therefore do not violate the rule that the validation data should only be used for model testing.

While hold-out validation is the most straight-forward approach, it works less well with small datasets (<100–200 samples) because uncertainty of the performance in the validation dataset will be large, and the diversity of the training data may be insufficient to discover a robust model. If obtaining more data is unfeasible, and in the case of smaller studies, cross-validation can be used to estimate performance.

With K-fold cross-validation, data are partitioned into K folds (typically 3–10), then K-1 folds are used to train the model, and the remaining fold is reserved to test the model. In this way, K separate models are trained, in which each fold plays the role of the test set. The final performance estimate is the average over all the folds, and the standard error of the performance can be estimated by using the standard deviation over the folds. This is useful when comparing different models and reflects the robustness of the model.

**Figure 7.**
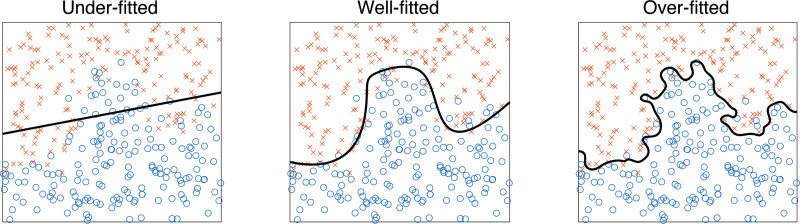
Example 2D classification tasks show the impact of under- and overfitting. In the case of underfitting, the linear model fits a straight line and does not have the capacity to capture the nonlinear (curved) nature of the decision boundary, and so its classification performance on both the training and the test data will be suboptimal. In the case of overfitting, the model is insufficiently constrained and tends to generate a complex decision boundary that is overly influenced by noise. In this case, the performance in the training data will be good but will worsen when evaluated on independent test data. Many machine learning models have tuning parameters that can be adjusted to give models at both ends of this spectrum, and so optimizing the tuning parameters (typically using cross-validation techniques) is necessary to produce a well-fitted model.

Many models have tuning parameters, and optimizing these parameters can be crucial for good performance. Unlike the model parameters, tuning parameters cannot be learned directly from training data. Poorly tuned parameters can lead to over- or underfitting of the training data—overfitting leads to poor performance in the validation data compared with the training data, and underfitting occurs when the model is unable to capture important features in the training data (Fig 7). Split-validation (equivalent to hold-out validation) and cross-validation can be used for optimizing the tuning parameters, and this enables tuning parameters to be found that balance between over- and underfitting. Similar validation approaches can be used to select between candidate models.

## Feature Stability

When manual segmentation is performed, it is important to reject radiomic features that are particularly sensitive to interreader variations in the ROI. This is evaluated by repeating tumor segmentations for a subset of patients by one or more readers. The intraclass correlation coefficient (ICC) can be used to reject nonreproducible features (35,36) below a threshold ICC.

While patients used for measuring reproducibility can be selected from the whole dataset, when hold-out testing is used it is convenient to select patients who are in the training data. In this case, the ICC threshold for feature rejection can be treated as a model parameter and optimized as a tuning parameter, but when this is not performed, ICC thresholds in the range of 0.75–0.9 are typical. Feature stability is also influenced by fluctuations in patient factors, including positioning; if possible, test-retest images on a subset of patients should be obtained. This is often feasible with MRI studies but can be more difficult for images obtained involving ionizing radiation and is usually not possible in retrospective studies.

## Univariate Feature Discovery

In radiomic studies, it is uncommon for a single feature to perform well enough to be used on its own, but univariate models (those that contain only one feature) are nevertheless useful as a benchmark baseline performance for comparison with more complex multivariate models (those that contain multiple features). For binary classification tasks (in which data are categorized into two groups [eg, benign vs malignant]) the area under the receiver operating characteristic curve is a suitable metric to rank the classification performance of each feature when used alone, and the Mann-Whitney *U* test can be used to test whether the model performs better than chance alone. As classification performance will be measured for each feature, multiple comparisons correction of the *P* values should be performed by using Bonferroni correction or false–discovery rate methods such as Benjamini-Hochberg and Benjamini-Yekutieli corrections (37,38).

## Feature Selection and Dimensionality Reduction

Multivariate models often perform better when feature selection or dimensionality reduction is applied because this tends to remove noise and reduces redundancy (the number of features that do not add any additional information to the model). A range of approaches are outlined in Table 5 and described in more detail in this section. A key consideration when choosing a feature selection technique is the impact on interpretability of the final model.

**Table 5:**
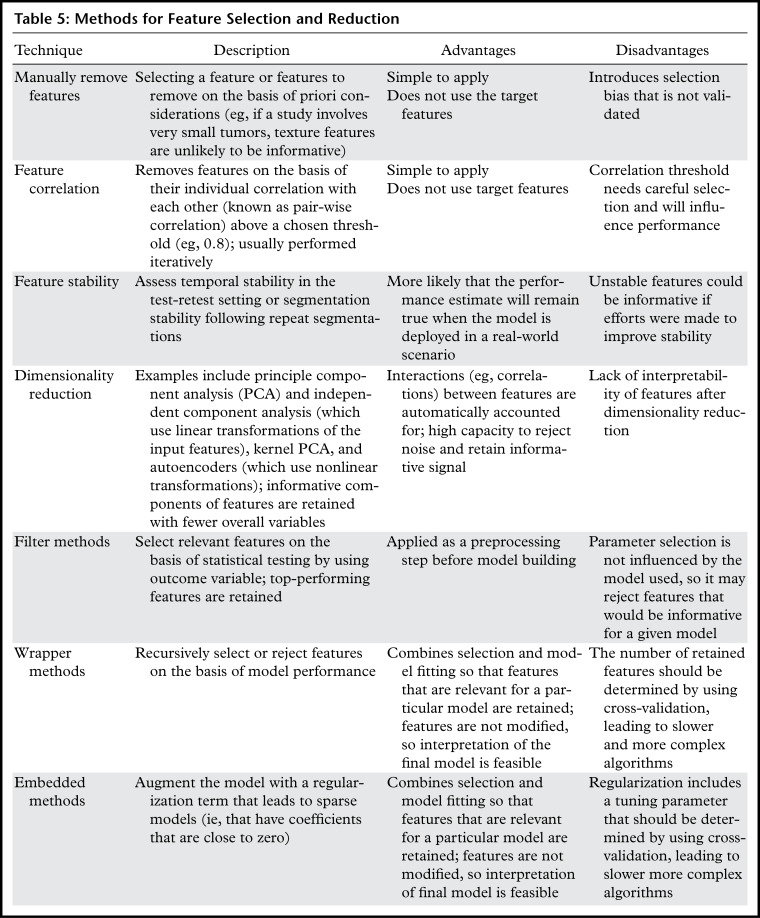
Methods for Feature Selection and Reduction

Correlated features can be reduced by using pairwise correlation statistics (such as Pearson correlation) to remove features that are correlated above a threshold (eg, 0.8). This is performed without knowledge of the outcome data and is done iteratively starting with the pair with the highest correlation. For each pair, the feature with the highest average correlation with the remaining features is rejected. To improve interpretability in addition to stability, we have developed an extension to this technique in which the classes of highly correlated features (ie, shape, first order, and texture) are used to determine which feature should be removed (15). For example, if a first-order and a texture feature are correlated, then the first-order feature is retained, and if a shape and a first-order feature are correlated, then the shape feature is retained. This results in a set of features with reduced redundancy and tends toward simpler interpretations.

Dimensionality reduction techniques aim to retain the informative components of features with a smaller overall number of variables. For example, the majority of “useful” information contained in 100 features is represented in one or two new variables that comprise combinations of the features. In this case, the dimensions have been reduced from 100 to one or two. Widely used examples include principle component analysis (PCA), independent component analysis, kernel PCA (39), and autoencoders (40). A key limitation is that variables obtained following feature reduction suffer from limited explainability since they are influenced by a combination of many or all of the input features.

Feature selection methods make use of the target data, and these can be divided into three types: filter, wrapper, and embedded methods.

Filter methods use statistics derived from each input feature and the target data to rank and select the input features and are applied to the training data before the model fitting. They are supervised (as they make use of the target data), and care should be taken to avoid data leakage from the validation data. Possible statistics include the *t*-statistic, Mann-Whitney *U* test, Fisher score, joint mutual information, maximum relevancy minimum redundancy, and mutual information (41).

Wrapper methods combine the chosen multivariate model with a feature ranking function that is used iteratively to remove low-ranking features. To avoid overfitting, the ranking should be computed by using cross-validation or split validation on the training data. Recursive feature elimination is a popular wrapper method and is available in most statistical packages.

Embedded methods take an existing statistical model (eg, logistic regression) and add a term (known as the regularization term) that has the effect of shrinking model parameters that are associated with noninformative features to values at or near zero. This simplifying property is advantageous when attempting to interpret the final model. Examples include Least Absolute Shrinkage and Selection Operator (LASSO) (42), ridge, and elastic net regularization (43). Embedded methods have one or more tuning parameters, and these should be optimized in the training data by using cross-validation or split-validation.

## Multivariate Models

Multivariate models refer to those that use multiple input variables and are frequently used in radiomic studies. The workhorse models for radiomics studies are classification and time-to-event (survival) models (Table 6).

**Table 6:**
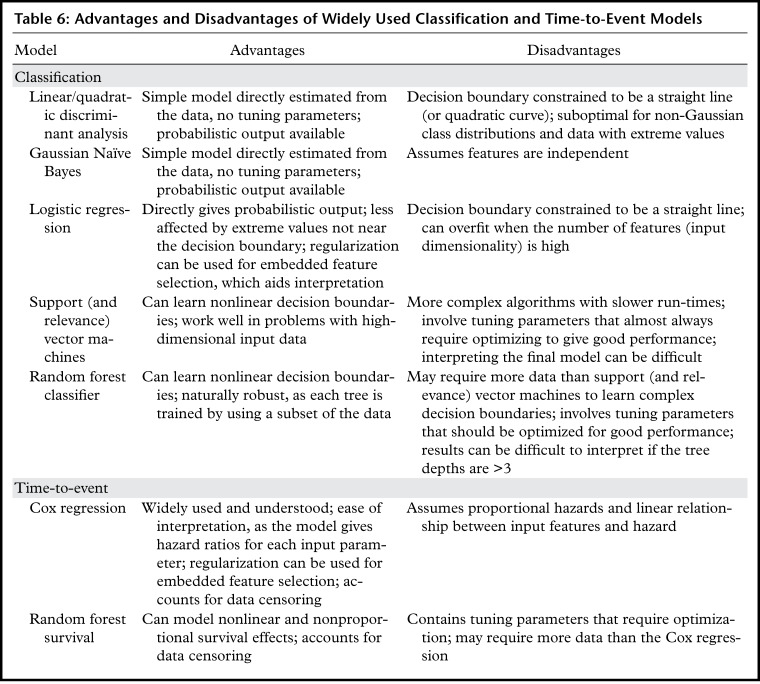
Advantages and Disadvantages of Widely Used Classification and Time-to-Event Models

Classification models generate boundaries between the data to separate them into discrete groups (Fig 7). These are referred to as decision boundaries, and data are classified on the basis of which side of the boundary they are located. A widely used group of classification models generate linear boundaries (ie, a straight line) or quadratic boundaries (a curve). These include linear discriminant analysis (LDA) and Gaussian naïve Bayes and quadratic discriminant analysis. Logistic regression is a related technique that (like LDA) generates a linear decision boundary, but unlike LDA, data points that are far from the boundary have a reduced effect on the location of the boundary. These classification models have the advantage that they do not have any tuning parameters but the disadvantage that they can only generate linear (or quadratic) decision boundaries, which may result in underfitting if the true boundary separating classes is not simply a straight line or quadratic function. These techniques can be used in combination with all three feature selection methods described previously. Logistic regression with LASSO regularization is a widely used example of this and has the advantage that the model parameters can be interpreted as odds-ratios, and the regularization tends to remove noninformative features, which aids model interpretation.

When the data require a more complex decision boundary, nonlinear classifiers such as support vector machines, relevance vector machines, random forests, and neural network classifiers may be appropriate (33,44). These algorithms can generate more complex boundaries between classes (compared with those of a linear or quadratic function) and have tuning parameters that can have a dramatic effect on performance, and so cross-validation or split-validation on the training data should be used for tuning parameter optimization.

Evaluating the performance of classification models learned from data is a crucial aspect of the development of a radiomic signature. Some of the more widely used metrics and their uses are outlined in Appendix E2.

Time-to-event models widely used in radiomics studies include Cox regression and random forest survival models. Both models account for data censoring. In radiomic studies with a large number of input features, Cox regression with LASSO regularization can be effective at generating a risk signature with a small number of nonzero features (45). Performance assessment of time-to-event models broadly falls into two types: prediction accuracy at a given time point or accuracy at predicting risk for the whole survival curve (46). Common metrics for assessing these are outlined in Appendix E2.

## Software

The main initial consideration when choosing radiomic software is whether to use commercial or noncommercial software. Noncommercial applications tend to be free, rapidly evolving, and reflective of the latest research trends. Commercial applications are not free but may be more stable, come with technical support, and are potentially a “black box.” As with all scientific software, users should consider the maturity level of the chosen package, documentation available, previous use in the literature, and potential for support from the individual or organization developing it. Additional radiomic-specific considerations include picture archiving and communication system (PACS) integration, segmentation tools, radiomic features supported, preprocessing, and model building. If local expertise is available, consider implementing an in-house pipeline that may be optimized to local systems. A large number of noncommercial software applications (Appendix E3) have been developed, and many are freely available for public download.

At present, there is limited choice of commercial software in this rapidly developing field. This likely represents a combination of the low potential for revenue where many open-source solutions already exist and the high barrier for developing software as a medical device for sale to the health care market. It is important to note that reproducibility is not guaranteed simply by using IBSI-tested software but also relies on harmonizing certain settings (which do not necessarily correspond to the defaults of the software) and maintaining consistency in versions of each software platform (47).

## Manuscript Writing

The interpretation of findings from radiomic studies requires detailed knowledge of the various steps performed during the study design, and it is crucial that these are clearly outlined when preparing a manuscript. To aid authors and to provide a framework for manuscript writing, there are various radiomic- and artificial intelligence–specific checklists, reporting guides, and radiomic quality scores that can be referred to (4,21,48,49), in addition to artificial intelligence extensions of familiar guidelines such as TRIPOD (Transparent Reporting of a multivariable prediction model for Individual Prognosis Or Diagnosis) (50), CONSORT (Consolidated Standards of Reporting Trials) (51), and SPIRIT (The Standard Protocol Items: Recommendations for Interventional Trials) (52).These can help assist with manuscript preparation, with insights into how manuscripts will be assessed at peer review. To address the challenge of standardization in radiomics, it is important to observe recognized nomenclature, for example, that collated by the IBSI (21).

Processing and acquisition parameters should be specified for all stages of the study, in addition to software details and version numbers. It has been proposed that liberal use of supplementary materials to include imaging protocols, examined images, segmentations, formulas for feature extraction, and code of radiomic models is encouraged (4). Where it is not possible to present patient-specific data, computed values from a digital phantom (53) can be used and compared with validated tolerance levels (54).

## Future Directions and Challenges

Although there has been an exponential increase in the number of radiomic publications, routine clinical implementation is yet to occur (55,56).

Key obstacles include noncompliance with machine learning best practices, standardization of the radiomic workflow, and clear reporting of study methodology. Only then can models be validated, preferably prospectively on external real-world data, including multivendor images and a variety of acquisition protocols.

Data curation and quality and adequate sample sizes are crucial in meeting these challenges. However, curation of large datasets is resource intensive, and accrual of sufficient data from multiple institutions can be challenging. Data sharing can help address these challenges. However, hurdles remain (57), including but not limited to ethical and legal considerations, data value, intellectual property, and resource availability.

Finally, although radiomics is largely a data-driven exercise, a deeper understanding of the biologic meaning of any derived radiomic signatures is required before results gain wider acceptance (6).

## Conclusion

Radiomic applications in oncology include diagnosis, prognostication, and prediction of clinical outcomes. It is a multidisciplinary field, encompassing radiologists and data and imaging scientists. A variety of challenges exist, including a need for standardization across all stages of the workflow and prospective validation across multiple sites using real-world heterogeneous datasets. This article provides multiple learning points to improve study design and execution and to enhance translation of radiomics into clinical practice.
